# Detection of Residual 2-Phenylphenol on Lemon Rind by Electrochemically Deposited Poly(hydroxybenzaldehyde) and Poly(hydroxybenzoic acid) Polymeric Stackings as Electrode Modifiers

**DOI:** 10.3390/ma16010357

**Published:** 2022-12-30

**Authors:** László Kiss, Zoltán Nagymihály, Péter Szabó, László Kollár, Sándor Kunsági-Máté

**Affiliations:** 1 Department of Organic and Medicinal Chemistry, Faculty of Pharmacy, University of Pécs, Honvéd Street 1, H-7624 Pécs, Hungary; 2 Green Chemistry Research Group, Szentágothai Research Center, Ifjúság útja 20, H-7624 Pécs, Hungary; 3 Environmental Analytical and Geoanalytical Research Group, Szentágothai Research Center, Ifjúság útja 20, H-7624 Pécs, Hungary

**Keywords:** hydroxybenzaldehydes, hydroxybenzoic acids, mesityl oxide, 2-phenylphenol, lemon rind

## Abstract

This study explores the characteristics of electrodeposition of the three hydroxybenzaldehyde isomers and selected hydroxybenzoic acids (4-hydroxybenzoic acid, salicylic acid, 3,5-dihydroxybenzoic acid) from mesityl oxide solvent. Similar to recent advances of this solvent, used by electrochemical studies, the carbon–carbon double bond had significant influence on the formation of polymers from the outlined molecules. In case of most substrates the peak currents increased to a steady-state but electropolymerization of some substrates caused significant deactivation. Scanning electron microscopic and complementary voltammetric studies facilitated that the electrochemically formed polymers are present on the electrode surface in stackings. In viewpoint of analysis of 2-phenylphenol, the modifying deposit formed from 4-hydroxybenzaldehyde was the best with 5 µM detection limit obtained with differential pulse voltammetry. Furthermore, a new procedure was chosen for the involvement of a cavitand derivative into the organic layers with the purpose to improve the layer selectivity (subsequent electrochemical polymerization in an other solution). Further studies showed that in this way the sensitivities of as-modified electrodes were a little worse than without this step, thus indicating that application of this step is disadvantageous. Recovery studies of 2-phenylphenol were carried out on lemon rind without any treatment, and it was compared with the case when the outer yellow layer was removed by rasping. The inner tissues showed very high adsorption affinity towards 2-phenylphenol.

## 1. Introduction

In the last decades, electrodeposition of organic layers received a great attention and their electric and many other properties can be governed by the experimental conditions. The polymeric coverage on surfaces of the commonly used electrodes built by electrochemical polymerization are attractive due to different uses. This way, the development of these organic layers needs usually no additional chemical reagent but sometimes special solvent or electrolysis solution composition is necessary [1,2]. The modified electrodes are mainly utilized in electrocatalysis and analysis of selected compounds. In the latter application the surface modification contributes often to the better selectivity and sensitivity [3,4,5,6,7,8,9,10]. 

The electrochemical synthesis of the modifying organic films can be conducted by controlled parameters but of course the appropriate technique should be applied to obtain the desired film composition. Therefore, the potentiostatic mode is a very familiar approach in this viewpoint where a steady potential is imposed to the working electrode. The other popular technique is cyclic voltammetry where the cycling between two potentials is carried out more times and within the potential range the desired process takes places on the electrode. The thickness of film can be controlled in potentiostatic mode by setting the time of deposition. However, when cyclic voltammetry is used the process can be regulated by proper choice of cycle numbers and scan rate. Due to the advantages of the latter method, cyclic voltammetry is applied in the most cases for electrodeposition. The thickness of polymers prepared with the above methods are usually grown to some hundreds of nanometers with some porosity. The analytes can easily diffuse through the pores but their size depends on the experimental conditions of deposition. The usefulness of the organic layers highly depend on the nature of matrix containing the analyte(s). For example, aqueous solutions cannot enter the pores of highly hydrophobic layer thus preventing the recording of current signal of the analyte(s). 

Although electronically conducting polymers have gained significance of electroanalysis [11,12], the high capacitive currents make their use disadvantageous. The low capacitive currents are characteristic typically for the phenol-based polymers so this is one of the causes of their attractive use. The phenolic monomers are widespread in sensor development and deposition of molecularly imprinted polymers (MIPs) has many analytical advantages [13,14,15,16,17,18]. The first step is during electrooxidation of phenols generally the formation of a phenoxyl radical which then couples with other electrogenerated radicals forming a coherent layer of the corresponding poly(phenyleneoxide) [19,20]. 

Recently published works showed that mesityl oxide used as solvent has favorable properties for organic electrodeposition reactions [21,22]. This solvent was used by our research group for electrodeposition studies of organic monomers susceptible to polymerization through formation of electrogenerated radicals. In fact, a copolymer will be the product of these reactions taking place in mesityl oxide due to the carbon–carbon double bond of solvent also when an electroactive substrate is present in solution. The polymers formed in mesityl oxide showed advantageous properties in electrochemical analysis compared with methyl isobutyl ketone possessing very similar chemical structure as in this solvent organic deposits formed predominantly with tortuosity governed properties [23].

There is a class of macromolecules where aromatic molecules are interconnected forming a cavity, which is usually four or six membered. These cyclic oligomers are named as cavitands. Multiple methods showed that these cyclic oligomers can bind aromatic or unsaturated bond bearing compounds through *π*–*π* weak interactions [24,25,26,27,28]. This aromatic skeleton is in most cases electron-rich so accommodation of electron-poor guest molecules is favored in it. 

The focus of this work is the investigation of electrodeposition of polymers of hydroxybenzaldehydes and hydroxybenzoic acids from mesityl oxide and assessing their ability to signal enhancement in determination of 2-phenylphenol. Especially hydroxybenzaldehydes are poorly investigated in respect of electropolymerization. Special emphasis was taken on the pretreatment of lemon rind by comparing the adsorption capabilities towards the chosen electroactive analyte.

## 2. Materials and Methods

All chemicals utilized for the experiments were analytical reagent grade and used without further purification. A platinum disc of 1 mm in diameter was the working electrode during the entire experimental procedure, which was connected to a platinum wire counter and silver wire reference electrode. The microdisc was sealed in polyetheretherketone as insulating sheath. This three-electrode cell was connected to a potentiostat (Dropsens, Oviedo, Spain). Before the studies a cleaning procedure was applied consisting of polishing with aqueous suspension of alumina on a polishing cloth (eDAQ) and ultrasonication in deionized water. The final cleaning step was the thorough rinsing with deionized water and dry acetone. The dry acetone removed the water traces from the working electrode as electrochemical behavior of the studied compounds are susceptible to the presence of water. For visualization of deposits a Jeol JSM-IT500HR (Jeol, Tokyo, Japan) scanning electron microscope (SEM) was used in the secondary electron mode and a 30 kV acceleration voltage was applied. The platinum electrodes used for microscopic visualization were carefully cleaned by polishing and then by ultrasonicating for 10 min in doubly deionized water, finally dried with thorough washing with acetone. The electrochemically prepared modifying layers were washed with acetonitrile to remove the unreacted species and supporting electrolyte and this solvent did not dissolve the formed polymeric products.

The lemon was purchased from a supermarket as real sample for 2-phenylphenol determination, and where it was necessary, the rind was removed by rasping with a household rasp. 

## 3. Results

### 3.1. Study on Electrodeposition of Organic Substrates from Mesityl Oxide and Surface Characterizations

**Figure 1:**
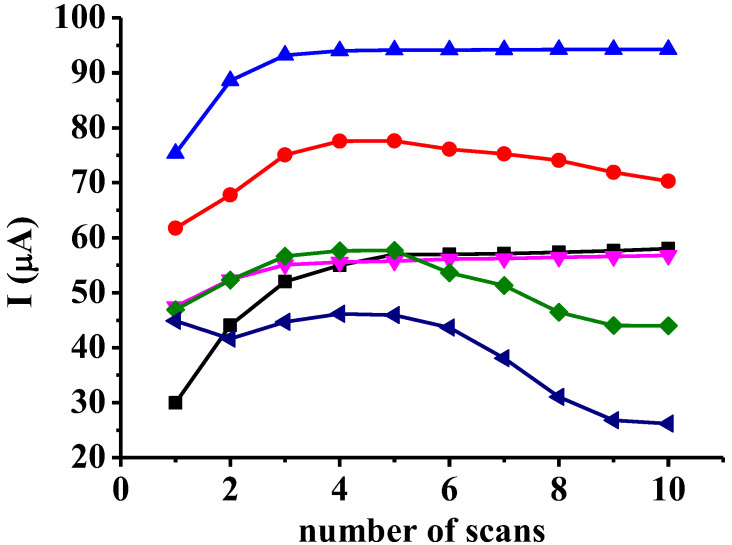
Peak currents of the ten subsequent cyclic voltammograms of 2-hydroxybenzaldehyde (■), 3-hydroxybenzaldehyde (●), 4-hydroxybenzaldehyde (▲), 4-hydroxybenzoic acid (▼), salicylic acid (♦), 3,5-dihydroxybenzoic acid (◄) (c = 50 mM, supporting electrolyte 50 mM TBAP).

In the first part of investigation the selected monomers were studied in mesityl oxide taking ten subsequent voltammograms with each one between 0 and 2.5 V with 0.1 V/s scan rate (Figure 1). It is remarkable that in the first cycles the majority of monomers show similar behavior to the cases when a conducting polymer is deposited as the peak currents increase but not this is the case. Then, the uniformity of peak heights from approximately the fourth scan suggests that by some substrates stationary diffusion occurs through the growing film. On the other hand, favorable solvation properties of the polymers make possible the enhanced accessibility of platinum surface for the monomer molecules. Similar observations highlighted earlier that it is characteristic for mesityl oxide solvent. In other common organic solvents, such as acetonitrile, the continuous current decline could be observed by studying the underlying monomers. Two hydroxybenzoic acid monomers showed predominantly tortuous behavior, salicylic acid and 3,5-dihydroxybenzoic acid attributable to the position of substituents on the benzene ring. Para position relative to the phenolic hydroxyl group is the most favorable for coupling of an other electrogenerated radical. This position is occupied by 4-hydroxybenzoic acid and together with the carboxyl group the forming polymer cannot engage a plain position on the electrode. As a consequence, this leads to weaker adherence of macromolecules. Significant role of carboxyl groups can be attributed to it as they associate with each other readily in aprotic environments. 

In these experiments, insulating polymers form and therefore the peak currents should continuously decrease, but the increase during layer preparation can be observed in accessibility of platinum surface for the substrate molecules. This effect is facilitated by polymer swelling taking place during the anodic polymerization process, which is usually characteristic for ketone solvents. 

**Figure 2:**
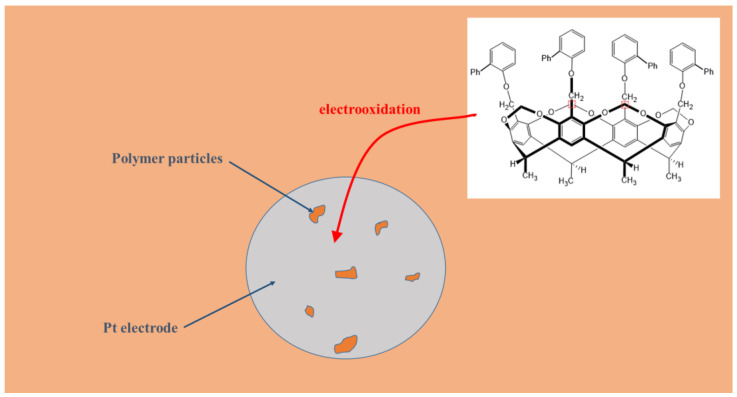
Schematic of building up the modifying organic layer on platinum electrode.

A cavitand bearing four 2-biphenyloxy moieties at the upper rim was used to alter the properties of the previously deposited phenolic compounds shown in the schematic graph of the process (Figure 2). In a recent work this cavitand was copolymerized in mesityl oxide with the phenylphenol isomers [23]. The co-deposited polymer in this way showed only a subtle improvement in increase of sensitivity compared with films deposited without this cavitand. A possible reason was for it the entrapment of segments of poly(phenylphenol) molecules into the cavity of the cyclic oligomer. Herein, the film modification with the cavitand was carried out with its subsequent polymerization by incorporating its products in the polymer layer of the simple phenolic compounds. 

**Figure 3:**
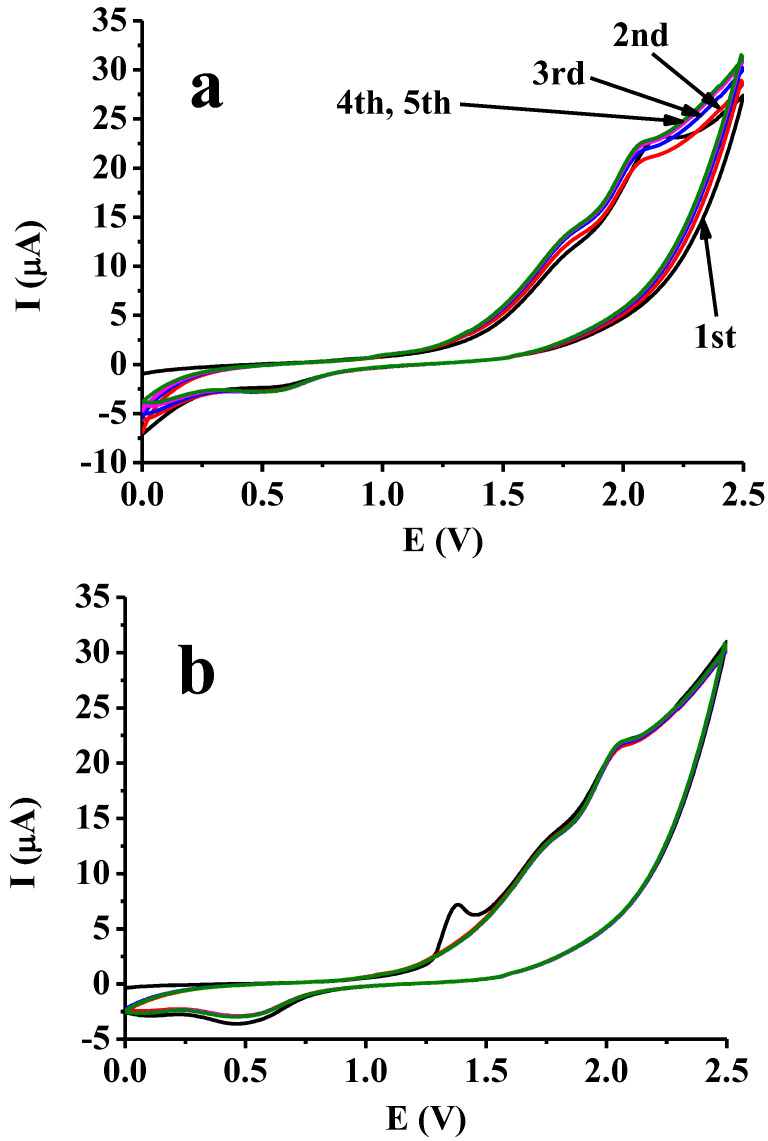
Cyclic voltammetric curves of 5 mM cavitand in mesityl oxide (**a**) and after deposition of poly(4-hydroxybenzaldehyde) (**b**).

The cavitand itself was also studied in mesityl oxide with cyclic voltammetry (Figure 3a) but because of the high background currents of solvent at higher potentials only a small peak appeared attributable to the cavitand at approximately 2.1 V. When this cavitand was examined on modified electrode with previously deposited poly(4-hydroxybenzaldehyde) this peak was very small and the voltammograms were uniform in viewpoint of magnitude of currents and similar to the case when only the cavitand was present in solution (Figure 3b). Basically, the shape of curves were identical in both cases. The first voltammogram in the second case contains a visible peak at approximately 1.35 V attributable to the poly(4-hydroxybenzaldehyde) deposit. When 4-hydroxybenzaldehyde was electrodeposited from mesityl oxide this peak also appeared in the subsequent scans and its height grew continuously to a limiting value (not shown). This suggests a partial electroreduction of the organic layer and its reoxidation occurs when the anodic peak appears. The reason for why this peak does not appear in the further scans in solution containing only the cavitand is the removal of redox active functional groups from electrode (see in the next paragraph). 

**Figure 4:**
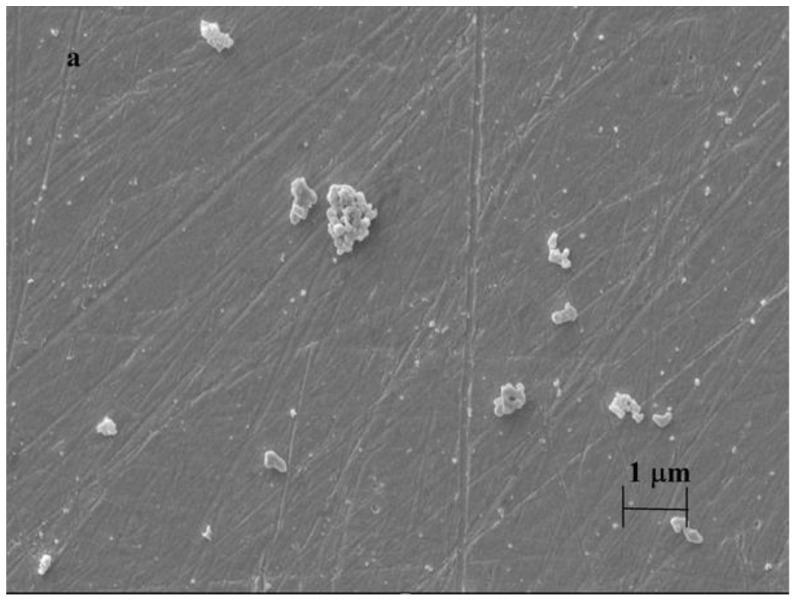

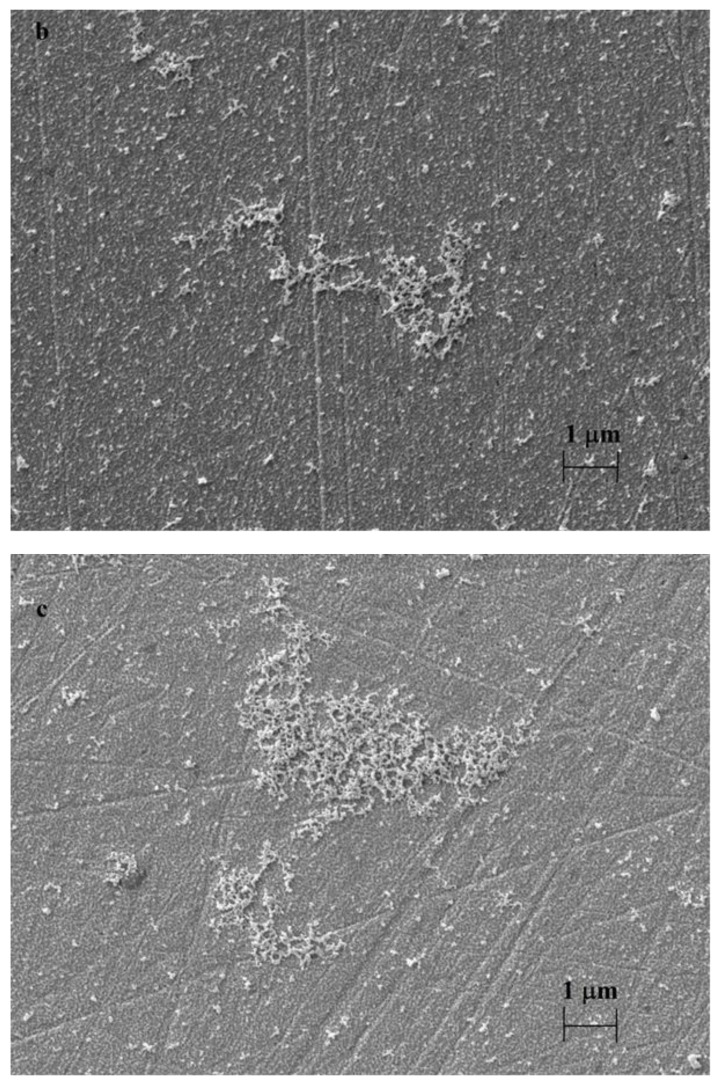

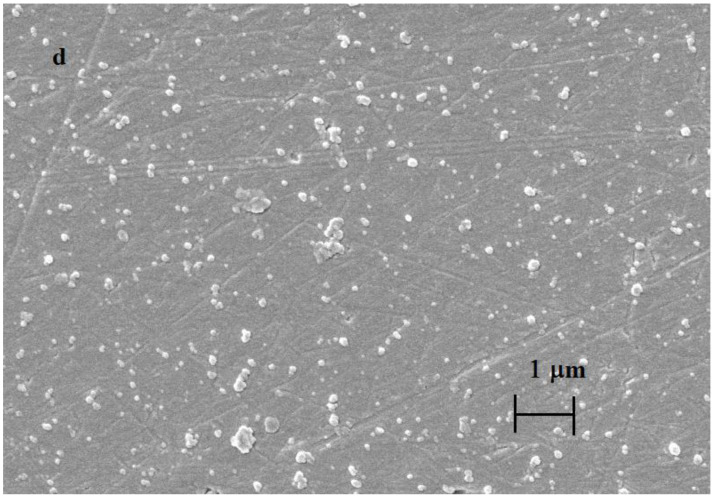
Scanning electron micrographs of modified platinum electrode surfaces (**a**): poly(4-hydroxybenzaldehyde), (**b**): after scanning in the 5 mM solution of cavitand, (**c**): after scanning in the solution of cavitand with the previously modified electrode with poly(4-hydroxybenzaldehyde)), (**d**): poly(4-hydroxybenzaldehyde) after 30 cycles.

The electron micrographs of deposits are displayed in Figure 4 and remarkable differences arise from application of the cavitand. When only 4-hydroxybenzaldehyde was electrochemically polymerized in mesityl oxide stackings developed on the surface of platinum. Involvement of the cavitand in the procedure resulted the formation of many small islands and moreover, the stackings were not at all observed characteristic of poly(4-hydroxybenzaldehyde) in spite of that 4-hydroxybenzaldehyde was previously deposited. The image is practically identical to the case when only the cavitand underwent anodic oxidation. These observations suggest that the stackings will be removed if the electrode is kept too long in the solvent. Part d reveals the electrodeposited particles from solution of 4-hydroxybenzaldehyde and the only difference was in the conditions compared with that of part a that 30 cycles were carried out in the same solution instead of 10. This shows that the increasing of electrolysis time leads to the parallel formation of more particles and the majority of them stays on the surface of electrode bound by weak interactions. 

Although the micrographs show that the electrodeposited polymeric materials are concentrated in stackings this itself cannot give evidence completely for layer formation. As the outlined polymers are hydrophobic, they keep the aqueous solutions away from the electrode surface where they cover it as it was found in a recent work [23]. So there is a simple and fast method to decide whether coherent film forms at the electrode. This is the comparison of current peak height of modified electrode with that of bare electrode in an aqueous solution of a redox active material. Now, a 5 mM solution of potassium ferricyanide was chosen and with the modified electrode 83% of current peak height obtained with bare electrode could be recovered. This value was obtained after background correction used usually when voltammetric studies are carried out [29], and this gives information about the surface coverage of electrode, and it can be concluded that nearly the whole part is free for electroactive materials. In fact, there are mainly particles of polymeric products facilitating the observations with microscopy. This is a high difference between films investigated here and the recently examined poly(phenylphenols). 

### 3.2. Electrodeposition of Organic Substrates from Mesityl Oxide and Assessment of Their Deposits as Modifier in Detection of 2-Phenylphenol

Further experiments aimed at assessing the polymer films in electrooxidation of 2-phenylphenol. The polymers formed from aromatics have usually high affinity towards analytes belonging to the aromatics due to the relatively strong secondary *π*–*π* interactions. 2-Phenylphenol is a widely investigated compound for multiple reasons, therefore, of all phenylphenol isomers its occurrence is far the highest in our environment. It is a powerful fungicide so its application is wide in storage and transport of vegetables and fruits and therefore its determination was aimed especially in citrus fruits [30]. In natural environments 2-phenylphenol can be produced by microbial and photolytic degradation processes, which help decomposition of phenoxyalkanoic and organophosphorous chemicals [31]. 

**Figure 5:**
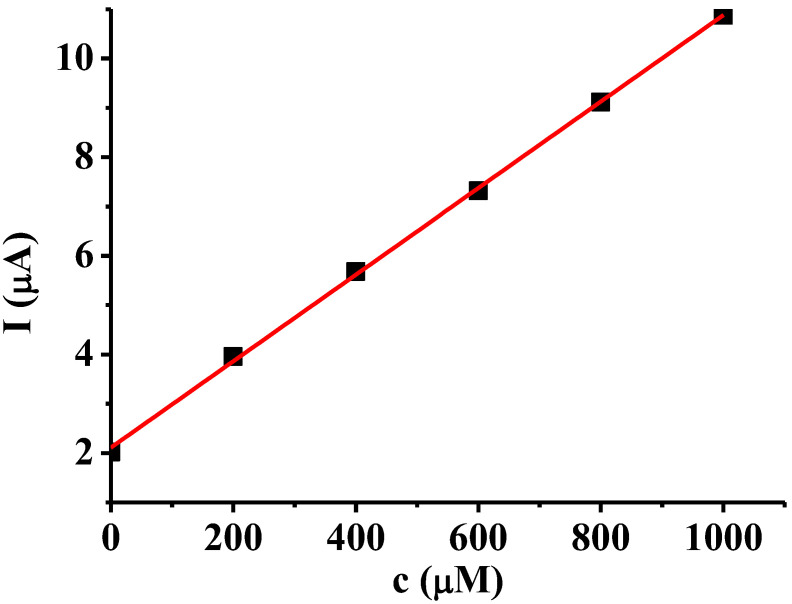
Linear sweep voltammetric peak currents versus 2-phenylphenol concentration in acetonitrile as calibration curve with bare platinum electrode (scan rate 0.1 V/s, supporting electrolyte 10 mM TBAP).

A 1 mM solution of this analyte was prepared with acetonitrile solvent. The linear sweep voltammetric peaks recorded between 0 and 2 V were used for analytical evaluation normalized to the anodic peak height obtained with the bare electrode. Before these studies the dependence of peak currents on the analyte concentration had to be established with bare platinum electrode. The calibration curve of 2-phenylphenol is linear in acetonitrile (Figure 5), indicating that at a concentration of 1 mM there is not any complication that could disturb the analytical procedures. This is important as concentrations in the linear range can only be used for assessing of deposits and 2-phenylphenol is susceptible to polymerization during its electrooxidation. The used solutions were dilute enough to avoid complications arising from deposit formation. The recoveries are collected in Table 1 for polymers prepared only with the use of phenolic monomers in mesityl oxide and for these polymers with subsequently deposited cavitand. The results clearly show that subsequent scanning in the mesityl oxide solution of the cavitand has disadvantageous effect on the analytical application. Poly(4-hydroxybenzaldehyde) seemed the most promising and the predominant role of tortuous effects were observed by the most hydroxybenzoic acid polymers in accordance with the previous deposition studies. Where signal enhancement was observed the stackings could adsorb 2-phenylphenol molecules. This binding is based on secondary interactions between aromatic moieties found in 2-phenylphenol and in the deposits. From the earlier studies a question arises whether the increasing of voltammetric cycle number (electrolysis time) improves the sensitivity of modified electrode towards 2-phenylphenol. The deposit prepared from 4-hydroxybenzaldehyde with 30 cycles was also tested. There was not considerable elevation in sensitivity only by approximately 1%. 

In a previous section it was shown that of the all studied deposits poly(4-hydroxybenzaldehyde) proved the best signal enhancement. Between 0 and 2.5 V cyclic voltammograms were taken in 50 mM solution of 4-hydroxybenzaldehyde prepared with mesityl oxide and after thorough washing with acetonitrile the modified electrode was dried. In this state it was ready for use. As 2-phenylphenol is widely used on the rinds of citrus fruits this work will focus on its differential pulse voltammetric determination. The maximum allowed concentration of 2-phenylphenol on citrus fruits is 12 mg/kg of whole fruit [32] so the chosen concentration range was adjusted to it. Other methods have been used also for the determination of 2-phenylphenol which have very high sensitivities, such as chromatography, fluorescence and spectrophoshorimetry [33,34,35,36], our method (as usually electrochemical methods) do not need large instrumentation. There are many possibilities for electrode modification to increase sensitivity and selectivity [37,38,39]. In this work a polymer particle modified electrode is tested.

**Figure 6:**
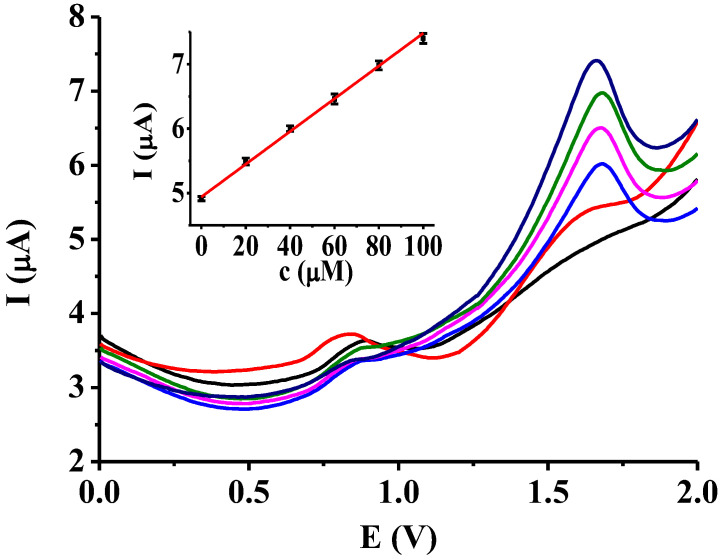
Differential pulse voltammograms of 2-phenylphenol with poly(4-hydroxybenzaldehyde) polymer particle modified platinum electrode (E_puls_ = 0.25 V, E_step_ = 0.004 V, t_puls_ = 5 ms, scan rate 0.04 V/s, supporting electrolyte 1 mM TBAP).

The concentration range was between 0 and 100 ∝ M using acetonitrile as solvent and 1 mM TBAP supporting electrolyte. The reason for why acetonitrile was used as solvent are the previous microscopic results as mesityl oxide removes the modifying materials so use of ketone solvents was inappropriate for accurate measurements. The potential window was between 0 and 2 V with the optimized measuring parameters. As shown in Figure 6, linear dependence was between peak current and concentration in the selected concentration range. The calibration equation is I(µ A) = 5.17 + 0.02c(∝ M) with R^2^ = 0.9953. The detection limit of the modified electrode was 5 µM using the 3σ method. The error bars in the calibration curve show that the measurements were well reproducible. A lemon served as the real sample in untreated form and in the form after removal of its outer colored layer by rasping. The rind of citrus fruits includes the outer yellow layer and the white bitter underlayer and they may have different adsorption properties. 500 µL of 0.01 M acetonitrile solution of 2-phenylphenol was put onto the surface of lemon and after evaporation of solvent it was placed in a vessel and 50 cm^3^ of acetonitrile was added to it. After thorough washing in this solvent and stirring, 4 cm^3^ of the obtained solution was pipetted into a vial adding the necessary amount of TBAP supporting electrolyte to get 0.001 M concentration for it. This procedure was repeated with a grinded lemon. The differential pulse voltammograms were recorded with lemons treated with 2-phenylphenol and also in untreated state. The current differences were used for evaluation at the potential of appearance of anodic peaks to minimize the effect of interfering compounds. However, some components of the colored outer layer certainly dissolved into acetonitrile, especially terpentinoids, their presence did not lead to appearance of new differential voltammetric peaks and significant change in the background curve as it was demonstrated in a separate experiment without 2-phenylphenol. The water content of the non-aqueous solvent can rise up the background curve and modify the analytical parameters of the modified electrode the outer layer of the rasped lemon was dried before the investigations. Using the calibration curve from the current differences 84.19 ± 3.07% recovery of 2-phenylphenol could be determined for untreated lemon and 13.33 ± 4.22% recovery for lemon treated by rasping averaged for three parallel measurements. These results showed that 2-phenylphenol readily adsorb to the outer colored layer and it will be practically fully absorbed in the white tissue presenting underneath the unharmed outer layer also after extraction with acetonitrile. This is a significant difference as when during the harvesting and transportation the rind is injured mechanically and treatment with 2-phenylphenol comes before carrying it to the supermarkets, significantly lower amount of material can be determined. Separate cyclic voltammetric results with some millimolar concentrations showed also that the grinded rind itself containing the inner tissues can adsorb the majority of 2-phenylphenol, both in freshly rasped and dried state. 

## 4. Conclusions

Studies described herein showed that for determination of residual 2-phenylphenol on lemon rind by platinum electrode, modification with electrochemically deposited poly(hydroxybenzaldehydes) and poly(hydroxybenzoic acids) polymers is appropriate. Efforts aimed at improving the sensitivity of the layer formed by using a cavitand derivative with subsequent electropolymerization were unsuccessful. Further studies are planned to optimize the formation of cavitand–phenylphenol inclusion complexes. The results also showed that lemon rind is powerful in biosorption applications so this can be an additional topic for future work. 

## Figures and Tables

**Table 1:** Recovery data of peak heights of deposits towards 2-phenylphenol.

Monomer	Recovery (Deposit) (%)	Recovery (Deposit + Cavitand) (%)
2-hydroxybenzaldehyde	107.39	99.23
3-hydroxybenzaldehyde	90.92	99.78
4-hydroxybenzaldehyde	110.35	99.88
Salicylic acid	86.11	97.13
4-hydroxybenzoic acid	108.57	98.77
3,5-dihydroxybenzoic acid	107.18	97.61

## Data Availability

The data presented in this study are available on request from the corresponding author.

## References

[1] McCarley R.L., Eugene A.I., Royce W.M. (1991). Permeant molecular sieving with electrochemically prepared 6-nm films of poly(phenylene oxide). J. Phys. Chem..

[2] Silvana A., Daniel A., Omowunmi A.S. (2003). A new electrocatalytic mechanism for the oxidation of phenols at platinum electrodes. Electrochem. Commun..

[3] Hong Y., Yuanyuan S., Xinhua L., Yuhai T., Liying H. (2007). Electrochemical characterization of poly(eriochrome black T) modified glassy carbon electrode and its application to simultaneous determination of dopamine, ascorbic acid and uric acid. Electrochim. Acta.

[4] Hong Y., Yuanyuan S., Xinhua L., Yuhai T., Ailin L., Guangwen L., Wei L., Shaobo Z. (2007). Selective determination of epinephrine in the presence of ascorbic acid and uric acid by electrocatalytic oxidation at poly(eriochrome black T) film-modified glassy carbon electrode. Anal. Sci..

[5] Youli W., Liqiang L., Yaping D., Xiao L., Yuliang C. (2013). A glassy carbon electrode modified with poly(eriochrome black T) for sensitive determination of adenine and guanine. Microchim. Acta.

[6] Xiao L., Liqiang L., Yaping D., Zhangping K., Daixin Y. (2012). Simultaneous determination of L-cysteine and L-tyrosine using Au nanoparticles/poly-eriochrome black T film modified glassy carbon electrode. Bioelectrochemistry.

[7] Parisa S.D., Fahimeh J. (2016). Differential pulse voltammetric determination of nanomolar concentrations of antiviral drug acyclovir at polymer film modified glassy carbon electrode. Mater. Sci. Eng. C.

[8] Shang-Lin Y., Ting-Ching W., Shin-ichi Y., Helmut T., Wei-Bor T. (2021). Conjugation of polysulfobetaine via poly(pyrogallol) coatings for improving the antifouling efficacy of biomaterials. ACS Omega.

[9] Soo B.K., Jing Z. (1998). Poly(pyrogallol) film on glassy carbon electrode for selective preconcentration and stripping voltammetric determination of Sb(III). Anal. Chim. Acta.

[10] Fei W., ChangLong C., Bo Y., Baoxian Y. (2015). Simultaneous voltammetric determination of dopamine and uric acid based on Langmuir-Blodgett film of calixarene modified glassy carbon electrode. Sens. Actuators B Chem..

[11] Simonas R., Arunas R. (2021). Conducting polymers in the design of biosensors and biofuel cells. Polymers.

[12] Teasdale P.R., Wallace G.G. (1993). Molecular recognition using conducting polymers—Basis of an electrochemical sensing technology. Analyst.

[13] Shuhuai L., Jianping L., Qingyu L., Xiaoping W. (2015). Molecularly imprinted sensor based on electrochemiluminescence membrane for ultratrace doxycycline determination. Analyst.

[14] Sara D., Beshare H. (2019). Voltammetric sensing of minoxidil using a molecularly imprinted polymer (MIP)-modified carbon paste electrode. Chem. Pap..

[15] Shahrzad S., Navid N., Mehdi E., Mojtaba K., Mostafa A. (2016). An electrochemical nanosensor based on molecularly imprinted polymer (MIP) for detection of gallic acid in fruit juices. Food. Anal. Methods.

[16] Hélder S., Joao G.P., Júlia M.M., Subramanian V., Cristina D. (2014). MIP-Graphene-modified glassy carbon electrode for the determination of trimethoprim. Biosens. Bioelectron..

[17] Yarma A., Kurbanoglu S., Jetzschmann K.J., Ozkan S.A., Wollenberger U., Scheller F.W. (2018). Electrochemical MIP-sensors for drugs. Curr. Med. Chem..

[18] Simonas R., Arunas R. (2022). Development of molecularly imprinted polymer based phase boundaries for sensors design (review). Adv. Colloid Interface Sci..

[19] Guzel Z., Ekaterina G., Elvira Y. (2021). Electrochemical sensors based on the electropolymerized natural phenolic antioxidants and their analytical application. Sensors.

[20] Yuqing M., Jianrong C., Xiahoua W. (2014). Using electropolymerized non-conducting polymers to develop enzyme amperometric biosensors. Trends Biotechnol..

[21] Kiss L., Kovács F., Li H., Kiss A., Kunsági-Máté S. (2020). Electrochemical polymerization of phenol on platinum and glassy carbon electrodes in mesityl oxide. Chem. Phys. Lett..

[22] Kiss L., Kovacs F., Kunsági-Máté S. (2021). Investigation of anodic behaviour of phenylethers in non-aqueous solvents on platinum and glassy carbon electrodes. J. Iran. Chem. Soc..

[23] Kiss L., Nagymihály Z., Szabó P., Kollár L., Kunsági-Máté S. (2022). Anodic polymerization of phenylphenols in methyl isobutyl ketone and mesityl oxide: Incorporation of a cavitand into the layers formed for sensing phenols in organic media. Molecules.

[24] Vishwanath D.V., Ashwini K.S. (2007). Electrochemical behaviour of folic acid at calixarene based chemically modified electrodes and its determination by adsorptive stripping voltammetry. Electrochim. Acta.

[25] Fei W., Yanju W., Kui L., Baoxian Y. (2013). A simple but highly sensitive and selective calixarene-based voltammetric sensor for serotonin. Electrochim. Acta.

[26] Dong-Sheng G., Kui W., Yu L. (2008). Selective binding behaviors of *p*-sulfonatocalixarenes in aqueous solution. J. Incl. Phenom. Macrocycl. Chem..

[27] Tapan S., Sira S., Armando R., Ashok M. (2018). Single-walled carbon nanotube-calixarene based chemiresistor for volatile organic compounds. Electroanalysis.

[28] Diana M., Vladimir B., Farida G., Mohamed AM F., Sofia K., Alsu G., Ramil N., Svetlana S., Igor A. (2021). Azocalix[4]arene-rhodamine supramolecular hypoxia-sensitive systems: A search for the best calixarene hosts and rhodamine guests. Molecules.

[29] Nagaraj P.S., Lokesh V.S., Rajesh N.H., Sharanappa T.N. (2009). Electrochemical oxidation of loop diuretic furosemide at gold electrode and its analytical applications. Int. J. Electrochem. Sci..

[30] Li H., Li J., Meng D., Peng J., Qiao Q., Yang Z., Xu Q., Hu X. (2011). Sodium dodecyl sulphate sensitized electrochemical method for subnanomole level determination of ortho-phenylphenol at a novel disposable electrode. Sci. China Chem..

[31] Narang A.S., Vernoy C.A., Eadon G.A. (1983). Evaluation of Nielsen-Kryger steam distillation technique for recovery of phenols from soil. J. AOAC Int..

[32] Kolbe N., Andersson J.T. (2006). Simple and sensitive determination of o-phenylphenol in citrus fruits using gas chromatography with atomic emission or mass spectrometric detection. J. Agric. Food Chem..

[33] Bartels M.J., Brzak K.A., Bormett G.A. (1997). Determination of ortho-phenylphenol in human urine by gas chromatography mass spectrometry. J. Chromatogr. B Anal. Technol. Biomed. Life Sci..

[34] Reyes J.F.G., Martinez E.J.L., Barrales P.O.P., Diaz A.M. (2004). Continuous-flow separation and pre-concentration coupled on-line to solid-surface fluorescence spectroscopy for simultaneous determination of ortho-phenylphenol and thiabendazole. Anal. Bioanal. Chem..

[35] Prousalis K.P., Polygenis D.A., Syrokou A., Lamari F.N., Tsegenidis T. (2004). Determination of carbendazim, thiabendazole, and o-phenylphenol residues in lemons by HPLC following sample clean-up by ion-pairing. Anal. Bioanal. Chem..

[36] Capitan-Vallvey L.F., Deheidel M.K.A., Avidad R. (2003). Solid-phase spectrophosphorimetric determination of the pesticide o-phenylphenol in water and vegetables. Anal. Bioanal. Chem..

[37] Deepti R.K., Shweta J.M., Prabhu K.K., Narasimba H.A., Raviraj M.K., Nagaraj P.S. (2020). Development of a novel nanosensor using Ca-doped ZnO for antihistamine drug. Mater. Chem. Phys..

[38] Shikandar D.B., Shetti N.P., Kulkarni R.M., Kulkarni S.D. (2018). Silver-doped titania modified carbon electrode for electrochemical studies of furantril. ECS J. Solid State Sci. Technol..

[39] Nagaraj P.S., Shweta J.M., Sharanappa T.N. (2015). Electro-oxidation of captopril at a gold electrode and its determination in pharmaceuticals and human fluids. Anal. Methods.

